# Supplementary material for: Keeping Cats Safe at Home (KCSAH): Lessons Learned from a Human Behaviour Change Campaign to Reduce the Impacts of Free-Roaming Domestic Cats
Source: Animals (Basel). 2025 Dec 10;15(24):3554. doi: 10.3390/ani15243554 (PMC12730004; doi:10.3390/ani15243554)
Supplement: Supplementary file 1 [file animals-15-03554-s001.zip › animals-3998097-supplementary.pdf]

Table S1. Summary of caregiver profiles identified through COM-B segmentation and their implications for targeted messaging (adapted from Ma & McLeod, 2023).

Profile	Defining beliefs / attitudes	Capability (C)	Opportunity (O)	Motivation (M)	Example tailored message levers
**1. Freedom Defender**	Strongly believe roaming is natural and beneficial for cats; low concern about risks.	Low: lack knowledge of harms.	Adequate housing/outdoor access.	High motivation *against* containment.	Reframe welfare: show that indoor cats can still exercise choice and stimulation; highlight injury/disease risks of roaming.
**2. Tolerant Guardian**	Reject or minimize impact of cats on wildlife; see containment as unnecessary.	Moderate: understand logistics but deny ecological consequences.	Adequate.	Low motivation.	Use emotive storytelling (injured wildlife/cats) and trusted messengers (wildlife carers) to make impacts tangible.
**3. Laissez-faire Landlord (a)**	Want to contain but doubt they can manage their cat indoors.	Low: lack skills to provide indoor engagement.	Variable housing suitability.	Moderate: intention present.	Provide practical "how-to" guides, DIY activity ideas, success stories from similar households.
**4. Laissez-faire Landlord (b)**	See pros and cons of roaming; feel unsure or inconsistent.	Moderate capability.	Adequate.	Low–moderate motivation.	Use social norming ("most caregivers in your community…") and highlight peace-of-mind benefits (safety, fewer complaints).
**5. Conscientious Caretaker**	Already convinced of benefits; actively seeking strategies.	High capability.	Adequate housing support.	High motivation.	Reinforce choices with tips, community recognition (pledges, competitions), and promote adoption of containment tech

					(fencing, enclosures).
**6. Concerned Protector**	Strongly value both cat welfare and wildlife protection; act as early adopters.	High capability.	Strong networks and supportive housing.	Very high motivation.	Equip as peer ambassadors—share stories, model behaviors, advocate in councils and schools.
